# Effects of Different Diluents on Semen Quality of Hu Ram Stored at 4 °C

**DOI:** 10.3390/ani13182823

**Published:** 2023-09-06

**Authors:** Liuming Zhang, Yanhu Wang, Xiaomei Sun, Yan Kang, Tariq Sohail, Jian Wang, Yongjun Li

**Affiliations:** Key Laboratory for Animal Genetics & Molecular Breeding of Jiangsu Province, College of Animal Science and Technology, Yangzhou University, Yangzhou 225009, China; 18352767281@163.com (L.Z.);

**Keywords:** diluent, motility parameters, membrane integrity, acrosome integrity, ROS

## Abstract

**Simple Summary:**

Diluent plays an important role in sperm storage. The various components of the diluent provide nutrition for sperm survival, maintain the stability of the preserved environment, and prolong the survival time of sperm. However, for the preservation of semen at 4 °C, diluent formulations are currently lacking or existing diluent formulations are not sufficient. Therefore, the effect of four diluents on semen preservation was studied through an assessment of sperm motility and functional integrity. Diluent D (Tris–Fructose–Citric acid–Egg yolk) was found to greatly improve semen quality during storage at 4 °C.

**Abstract:**

This study aimed to investigate the effects of various diluents on the quality of *Hu* ram sperm stored at 4 °C. Semen samples were collected from three *Hu* rams and diluted with diluents A (Sodium citrate–Glucose–Egg yolk), B (Sodium citrate–Glucose), C (Fructose–Skimmed milk powder–Soy lecithin), and D (Tris–Fructose–Citric acid–Egg yolk). Total motility (TM), straight-line velocity (VSL), average path velocity (VAP), curvilinear velocity (VCL), average motion degree (MAD), acrosome integrity, membrane integrity, and reactive oxygen species (ROS) were evaluated. The results showed that diluent D had better preservation in terms of the sperm TM, VSL, VCL, VAP, MAD, and membrane and acrosome integrity. On the third day of the storage, the sperm PM of diluent D was higher than that of other diluents (*p* < 0.05). The ROS level of diluent D was lower than that of other diluents on the fifth day (*p* < 0.05). On the seventh day of the storage, the sperm TM in diluent D reached 50%, which was the highest in all diluent groups. On the seventh day of the storage, the integrity of the sperm membrane and the integrity of the acrosome of the sperm in diluent D were the highest in all diluent groups (*p* < 0.05). In conclusion, these results indicated that diluent D improved the semen quality during storage at 4 °C. In this study, diluent D was the best diluent formula for *Hu* ram semen stored at 4 °C.

## 1. Introduction

*Hu* sheep are a local breed under first-class protection in China and are also the only breed of sheep with lambs with white skin [1,2]. *Hu* sheep have excellent reproductive characteristics such as early sexual maturity and being highly prolific [3,4]. Therefore, improving the large-scale and standardized breeding level of *Hu* sheep can effectively increase farmers’ economic benefits and improve people’s living standards. However, the result of the preservation of ram semen is not satisfactory. In Bhalothia’s [5] study of the preservation of ram semen at 4 °C, the TM decreased to 30% on the third day of preservation. In a study of ram semen, O’Hara [6] found that after three days of storage, the fertilization rate plummeted to just reached 20%. According to Druart [7], the pregnancy rate was significantly lower after insemination with semen stored for one day. With the extension of storage time, the quality of semen will decrease, which will further reduce the pregnancy rate [8].

Semen preservation is very important to prolong survival time, expand semen volume, effectively contribute to promoting the reproductive potential of excellent males, and overcome geographical limitations [9]. Semen preservation mainly includes room-temperature preservation (15–25 °C), low-temperature preservation (0–5 °C), and cryopreservation (−196 °C) [10,11]. Compared with fresh semen, frozen–thawed semen will have reduced sperm motility and structural integrity [12]. Cervical insemination with frozen–thawed semen generally resulted in low pregnancy rates [13]. The total motility (TM) of sperm stored at room temperature will decline in a short time, and the storage time is too short to satisfy the demand for extensive usage of semen [14]. At present, sheep semen is mainly preserved at low temperatures. Selecting an efficient and stable diluent formula for sheep semen is a prerequisite to improving the effect of low-temperature semen preservation. Low-temperature preservation of sheep semen refers to the method in which semen is diluted according to a certain proportion and stored at 4 °C to extend survival time [15]. At low temperatures, the metabolic rate of sperm is slowed down, even in a dormant state, and the accumulation of metabolites decreases significantly [16,17]. When stored at 4 °C, the reproduction and growth of microorganisms in the preserved semen were also inhibited, improving the quality of low-temperature preservation of sheep semen [18]. It should be noted that one study found that slow cooling during low-temperature preservation of sheep semen is the key to ensuring semen quality, and a sharp decrease in temperature will cause a cold shock to the sperm [19,20].

Currently, there are still many shortcomings in the low-temperature preservation of sheep semen, such as the uncertainty of basic diluent, the short survival time of sperm, and poor quality of semen. With the diluent formula for low-temperature preservation of sheep semen, the effective survival time of the sperm is only about three days, far from meeting the practical production needs. As the diluent formula in studies of low-temperature preservation of sheep semen does not give good results at present, the diluent formula selected in this study is based on the diluent formula of other species that are better preserved at low temperatures. Therefore, it is very important to select an efficient and stable diluent formula for low-temperature preservation of sheep semen. In this experiment, four kinds of diluent formulas for the low-temperature preservation of sheep semen were selected, and the best diluent formula was designated through the detection of semen storage quality. The purpose of this research was to assess the effects of different diluent formulas on the quality parameters (TM, straight-line velocity (VSL), average path velocity (VAP), curvilinear velocity (VCL), average motion degree (MAD), membrane integrity, acrosome integrity, and reactive oxygen species (ROS)) of *Hu* sheep semen when stored at 4 °C. 

## 2. Materials and Methods

### 2.1. Animals and Semen Collection

Semen samples from three sexually mature and healthy *Hu* sheep (about three years old) were used in this research. After reproductive examination, there was no problem with the testes of the three rams, and the testicles were normal in size according to visual inspection. The rams were housed at the Experimental sheep farm of Yangzhou University (Jiangsu, China). The rams were free to drink water and provided with high-quality hay and concentrate.

A total of 60 ejaculates (20 ejaculates per ram) were collected from the three rams with an artificial vagina two times a week from February to April 2022. The collected semen was brought to the laboratory within 30 min. First, the collected semen was preliminarily selected, and samples with a milky white color and about 1 mL volume of semen were selected. The quality of semen was evaluated using a computer-aided sperm analyzer (CASA). In this study, only semen samples with a concentration ≥2.0 × 10^9^ sperm/mL, motility ≥ 80%, and normal sperm morphology ≥ 85% were used. To overcome individual differences and balance the sperm contribution, the checked semen was pooled together.

### 2.2. Diluent Preparation

Four different diluents, A, B, C, and D, were prepared on the basis of composition and dose as shown in Table 1. In the laboratory, analytical balances (XSE105DU, Mettler Toledo, Zurich, Switzerland) were used to accurately weigh reagents from different formulations. The weighed substances were dissolved in 100 mL of sterilized ultrapure water and stirred with a glass rod to fully dissolve. The diluent was filtered through a 0.2 µm filter membrane for later use. After filtration, 10% egg yolk was added to diluents A and D, and they were stored overnight in a refrigerator at 4 °C. The final diluent was the supernatant obtained after centrifugation at 12,000× *g* for 10 to 15 min.

### 2.3. Semen Processing

After mixing the semen, it was split into four equal fractions and diluted with A, B, C, and D to 2.0 × 10^8^ sperm/mL. Finally, the semen samples were wrapped with eight layers of cotton and kept in a 4 °C refrigerator. During the storage period, the plastic tubes containing the semen were gently shaken every 12 h to prevent sperm from depositing. In addition, semen quality parameters (TM, VSL, VCL, VAP, MAD, and membrane and acrosome integrity) were measured and analyzed once a day. The ROS level was measured on the fifth day.

### 2.4. Evaluation of Semen Quality

#### 2.4.1. Analysis of Sperm Motility Parameters

Sperm TM, VSL, VCL, VAP, and MAD were assessed using the CASA (ML-608JZ II, Mailang, Nanning, China) equipped with a warm stage. The software used by the CASA is an automatic analysis and detection system for animal sperm belonging to Mailang. The microscope used in the detection system is an ML-800 (Mailang, Nanning, China). The microscope used a Mailang conjugate high-contrast sperm optical imaging device (10× PH). The CASA software (ML-800II) captured 30 frames per second. The diluted semen was incubated at 37 °C for 4 min. Then, each semen sample was placed on the Mailang computer sperm counting board (ML-CASA20-4) and estimated at 37 °C. The counting board was 20 µm deep. At least 200 sperm cells were observed per slide. The TM represents the proportion of active sperm. The VSL represents the speed of the straight-line movement distance of the sperm head. The VAP indicates the movement speed of the sperm head along its spatial average trajectory. The VCL indicates the velocity of the sperm head along its actual walking curve.

#### 2.4.2. Analysis of Sperm Functional Integrity

The functional integrity of the sperm membrane was evaluated using HOST, as described by Wang Y [21]. It was evaluated every day. Measurement was carried out by mixing 20 µL semen with 200 µL hypo-osmotic solution. After incubation at 37 °C for 40 min, sperm swelling was observed under a 400× phase-contrast microscope. At least 200 sperm cells were observed on each slide.

The Coomassie brilliant blue stain was used to assess the integrity of the acrosome as described by Zhang L, and the Coomassie brilliant blue staining solution was prepared according to their method [22]. These parameters were assessed every day. Briefly, 50 µL of the preserved semen sample was fixed with 1 mL of 4% paraformaldehyde at room temperature for 10 min. The sample was centrifuged at 422× *g* for 5 min, the supernatant was discarded, and 10 µL was taken to make a smear. After air-drying, the smear was stained with Coomassie brilliant blue solution for 40 min and washed with distilled water. After natural drying, a minimum of 200 sperm cells on each slide were examined under a 1000× phase-contrast microscope. Sperm heads have two distinct varieties. The acrosome was intact if the sperm head appeared blue. The acrosome was not intact if the sperm head appeared unstained. At the same time, the spontaneous acrosome reaction (SAR) was evaluated in order to reflect the real influence of diluent on the sperm acrosome.

#### 2.4.3. Analysis of the ROS Level of the Sperm

The ROS level of the sperm was measured with an ROS Assay kit (Beyotime Institute of Biotechnology, Shanghai, China) according to the instructions. First, a volume of 100 µL diluted semen was mixed with 300 µL of 4 °C pre-cooled PBS and centrifuged at 422× *g* for 5 min. This procedure was repeated two more times to remove the supernatant. Second, 300 µL DCFH-DC working solution was added to the sperm. After staining at 37 °C for 30 min in dark conditions, the samples were centrifuged at 422× *g* for 5 min, the supernatant was discarded, and the samples were washed with PBS two times. Finally, a volume of 600 µL PBS was added to the sperm. In the negative control group, DCFH-DC working solution was not added. In the positive control group, when 300 µL of CFH-DC working solution was added, 0.3 µL of Rosup reagent was added at the same time. The level of ROS was expressed as absorbance at an excitation wavelength of 488 nm and an emission wavelength of 525 nm for a microporous multimodal detection system (PerkinElmer Inc., NYSE: PKI, Waltham, MA, USA). The experiment was repeated three times.

### 2.5. Statistical Analysis

Statistical analyses were performed using SPSS 24.0. The Shapiro–Wilk test was used to test whether the data obey a normal distribution. The data do show a normal distribution. Duncan’s multiple range tests using one-way analysis of variance procedures were used to compare the mean values of TM, VSL, VCL, VAP, MAD, membrane integrity, acrosome integrity, and ROS level between diluents within the same time points. Changes in variables across different time points in a group were assessed using repeated measures ANOVA to reveal time effects. The significance level of *p* < 0.05 was chosen unless otherwise noted. The results are presented as mean ± SD. All groups were replicated three times. 

## 3. Result

### 3.1. Effects of Different Diluents on the Sperm Motility Parameters

Table 2 shows the effects of several diluents on the TM and kinematic parameters of the *Hu* ram sperm during liquid storage at 4 °C. With the extension of storage time, sperm motility gradually decreased, and the sperm motility of diluent B and diluent C decreased more rapidly. On the third day of storage, the sperm TM in diluent C dropped to 0. On the seventh day of storage, the sperm TM in diluent B dropped to 0. After three days of preservation, the sperm TM in diluent D was the highest among all the diluents.

On the fifth and sixth days of preservation, the sperm VSL in diluent D was significantly higher than that in the other diluents (*p* < 0.05). On the fourth, fifth, and sixth day of storage, the VCL and VAP of sperm in the diluent D group was significantly higher than that in other diluents (*p* < 0.05). On the second day of preservation, the MAD of sperm in the diluent A and D groups was significantly higher than that in other diluents (*p* < 0.05). From day 3 to day 7, the MAD of the sperm in the diluent D group was significantly higher than that in the other diluents (*p* < 0.05).

### 3.2. Effects of Different Diluents on the Sperm Functional Integrity

**Figure 1:**
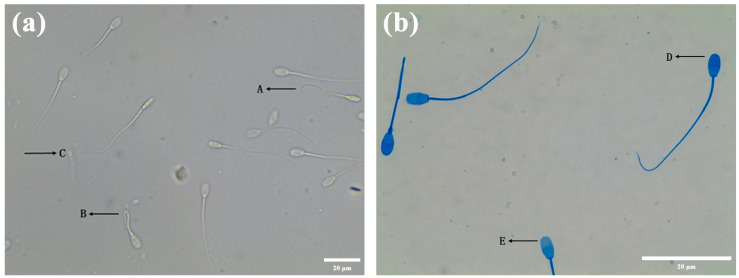
Detection of the sperm plasma membrane and acrosome. (**a**) The detection of sperm plasma membrane. The two types of tail curls A and B indicate intact-membrane sperm, and the tail non-curl type C is the sperm with a damaged membrane. (**b**) The detection of sperm acrosome. The sperm head is blue (D), which means that the acrosome is intact. The sperm head is unstained (E), which means that the acrosome is not intact.

The HOST microscopy result is shown in Figure 1a. There are three types of sperm tails. Sperm with a functional membrane are type A and B sperm, which have a curled tail. Sperm with a non-functional membrane are type C sperm, whose tail is not curled. Table 3 shows the effects of several diluents on the membrane function of the *Hu* ram sperm during liquid storage at 4 °C. From day 1 to day 7, diluent D was superior in terms of preserving the function of the sperm membrane during storage at 4 °C. These results indicated that diluent D was better preserving the membrane functional integrity of the *Hu* ram sperm during storage at 4 °C.

Figure 1b displays the result of the Coomassie brilliant blue staining. Table 3 shows the effects of several diluents on the acrosome integrity of the *Hu* ram sperm during liquid storage at 4 °C. The sAR of mixed semen was detected before semen diluents, and it was found that the value of SAR was only 4.55%, which was very low. The integrity of the acrosome of the preserved sperm in diluent C decreased the most rapidly. The integrity of the acrosome of the preserved sperm in diluent D was the highest among all the diluents (*p* < 0.05) on the seventh day of preservation.

### 3.3. Effects of Different Diluents on the Sperm ROS Level

**Figure 2:**
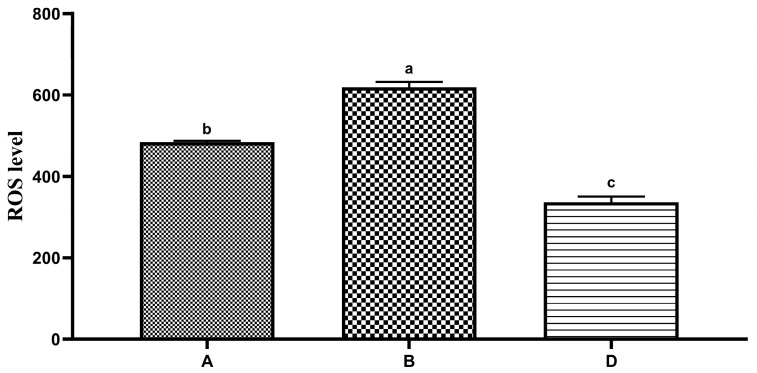
Effects of different diluents on the production of ROS in the sperm on the fifth day. Note: Different letters mean a significant difference (*p* < 0.05), but the same letter means no significant difference (*p* > 0.05).

The effects of different diluents on the ROS level of the *Hu* ram sperm during low-temperature preservation are shown in Figure 2. The ROS level of sperm in diluent D was significantly lower than that of the groups of diluent A and diluent B on the fifth day (*p* < 0.05). The ROS level of sperm in diluent B was significantly higher than that of the groups of diluent A and diluent D on the fifth day (*p* < 0.05).

## 4. Discussion

It is very important to detect the quality of semen before AI. Sperm motility parameters (such as sperm TM and kinematic parameters) and membrane and acrosome integrity are considered reliable indicators [23]. Therefore, in this study, the diluent formula suitable for the low-temperature preservation of *Hu* ram semen was identified by measuring the parameters of sperm TM, VSL, VCL, VAP, MAD, membrane integrity, acrosome integrity, and ROS level after different storage times. The experimental results show that diluent D has the best preservation effect. The sperm TM reached 51.01% on the seventh day after preservation and reached more than 86.44% on the fifth day. Followed by diluent A and diluent B, diluent C has the worst preservation impact on semen. On the fifth, sixth, and seventh s of preservation, the plasma membrane integrity and acrosome integrity of sperm in diluent D were significantly higher than those in other diluent groups. The basic diluent used by Merati [24] in the cryopreservation of sheep semen also contains the component of diluent D. After thawing with the optimized diluent formula, the sperm TM reached 45.93%. This study was consistent with Merati’s findings, and diluent D had effective preservation properties. Sperm motility is an important component of male fertility because of its importance for migration in the genital tract [25]. Therefore, the composition of diluent D could be suitable for the preservation of sheep semen at low temperatures and cryopreservation. Under two conditions of preservation, it had a favorable effect on the preservation. The addition and concentration of other substances in the formula may be related to specific sheep breeds and storage conditions. Before diluting the mixed semen with each diluent, the sAR of sperm was detected, and it was found that the sAR rate was extremely low. If the sAR rate is high, it will affect the quality of semen preservation and the subsequent implementation of AI technology [26]. This shows that diluent D can prolong the low-temperature preservation time of semen and is the best diluent formula for the low-temperature preservation of *Hu* ram semen.

Sugars play three main roles in the diluent. Carbohydrates are the main nutrient substance in the diluent, providing energy for sperm. Sperm can use monosaccharides, such as glucose and fructose or their metabolites, for glycolysis and oxidative phosphorylation to provide energy for metabolism and activity [27]. In a study of sheep sperm, Molinia [28] found that sperm motility in a diluted solution containing glucose and fructose reached 35.3% and in solutions containing sucrose and lactose reached 24.7% and 25.7%, respectively. This may be because the efficiency of sperm utilization of monosaccharides is higher than that of disaccharides. Therefore, simple sugars such as fructose and glucose may be added to sheep sperm to provide energy. In this study, the nutrients added to the four diluents were monosaccharides. Yildiz [29] found that the effect of adding fructose to sperm was that motility reached 84.3% and acrosome integrity reached 98.4%, which was superior to other monosaccharides. This study was consistent with Yildiz’s research results, and the sperm TM and acrosome integrity in diluent D were higher than that in diluent A. In addition to the effects of buffering substances, this may also be due to the higher utilization of fructose than glucose for sheep sperm.

At present, diluent containing milk, sodium citrate, and egg yolk is widely used in low-temperature preservation [30]. In this study, diluent A and D contained egg yolk, while diluent B did not contain any protective agent. The results indicated that the sperm TM in diluent A and D decreased slowly, while that in diluent B decreased rapidly. From the first day of semen preservation, the sperm TM in diluent A and D was significantly higher than that in diluent B. With the prolongation of semen preservation time, the sperm TM in diluent A and D was significantly higher than that in diluent B. On the seventh day after preservation, all the sperm stored in diluent B died, and the TM in diluent A and D, respectively, reached more than 30% and 50%. This suggests that egg yolk is essential to protect sperm from low-temperature effects. It may be that lecithin and unsaturated fatty acids in the yolk can alleviate the effect of free radicals, stabilize the cell membrane, promote lipoprotein synthesis, and improve sperm metabolism [31]. In addition, the addition of yolks can also improve the tolerance of sperm to osmosis. Egg yolk is a commonly used protective agent in low-temperature semen preservation diluents, although researchers suggest that adding egg yolk to the diluent may risk spreading animal diseases [32,33]. Some research is also devoted to the study of plant components that can replace egg yolks, such as soybean lecithin and others [34]. However, the egg yolk in the diluent can not only protect sperm from low-temperature effects but also provide nutrition for the sperm, which is not available in many protective agents [35]. Therefore, semen preservation still makes extensive use of egg yolks. In an experiment comparing the effects of untreated egg yolk and pasteurized egg yolk on semen, Selige [36] found that there was no significant difference in sperm PM, acrosome integrity, and membrane fluidity between the two groups, and the quality of sperm in both groups remained at a high level. This could be due to the fact that bacterial metabolism is effectively inhibited during cryopreservation, making any potential effect of bacterial metabolism unlikely. This study was consistent with Selige’s research results. Sperm TM, acrosome integrity, and functional membrane were higher in diluents A and D, supplemented with egg yolk, than in other diluents. This shows that the addition of non-sterilized egg yolk to the diluent may not necessarily lead to a decline in semen quality. Under the condition of the low-temperature preservation, the influence of bacteria may also be limited. Soybean lecithin is a permeable protective agent that is equivalent to a low-density lipoprotein in the yolk and has a beneficial effect on sperm [37]. Skimmed milk powder is an impermeable protective agent that plays a role in the regulation of osmotic pressure outside of sperm [38]. Diluent C added two types of protective agents: skimmed milk powder and soybean lecithin. However, the quality of semen after storage decreased rapidly, and all sperm died on the third day after storage, likely because no buffer solution was added to dilute it.

The buffer substance is also an important component of the diluent. Its main function is to buffer the acidic products produced by sperm metabolism during preservation, mainly lactic acid. Due to the prolongation of semen preservation time, acid substances gradually accumulate, which can cause sperm acidosis [30]. It can be seen that adding buffer substances to the semen diluent is of great significance to maintain sperm TM [39]. Currently, the buffer substances commonly used in diluents are sodium citrate, sodium dihydrogen citrate, citric acid, Tris, and some others. It is found that the buffering effect of Tris is very good, mainly because the effective range of Tris buffer is neutral, the toxicity to sperm is low, and it can have a better buffering effect, which is more conducive to the preservation of sperm [40]. Sodium citrate is an inorganic salt buffer that can also maintain the pH value of the diluent. Citric acid can maintain the pH value of the diluent, chelate heavy metals, and adjust the osmotic pressure [41]. It has been found that the effect of adding various buffers to the diluent is better than that of a single buffer [42]. Diluent D has the longest sperm storage time and can maintain high sperm quality. This may be because diluent D contains Tris and citric acid, while diluent A and diluent B contain only sodium citrate as buffer. The detection of ROS content also proves this. ROS originate from endogenous and exogenous sources. The mitochondrial oxidative respiratory chain of normal sperm produces endogenous ROS, but damage to the mitochondria results in the release of a large amount of ROS, and the released ROS continue to damage the sperm, causally linked in a repeated cycle that leads to sperm apoptosis [43,44,45], while exogenous ROS are produced as a result of harmful substances and environmental changes [46]. The level of ROS in diluent D was lower than that in other diluent groups on the fifth day. The pH of the diluent can be affected by the by-products of sperm metabolism. If the pH level exceeds a certain threshold, it can damage the structure of the sperm and induce the programmed cell death [47]. Tris and citric acid, contained in diluent D, are commonly used buffer substances that can keep the diluent stable and reduce sperm apoptosis. On the one hand, this may be because the buffer substance of diluent D is the most suitable and can keep the pH and osmotic pressure of semen relatively stable. On the other hand, the sperm in diluent B had the lowest TM and the highest number of dead sperm, which leads to a higher production of ROS by the apoptotic sperm. 

## 5. Conclusions

In this study, diluent D has a better low-temperature preserving effect on *Hu* ram semen, which can significantly improve the quality of semen preservation and prolong the semen preservation time. Comparing with other diluents, the best diluent formula suitable for the low-temperature conservation of *Hu* sheep semen was selected, which provided a theoretical reference for effectively prolonging the low-temperature preservation time of *Hu* ram semen and laid a foundation for AI technology. 

## Figures and Tables

**Table 1:** Four different diluent formulations.

Constituent	A	B	C	D
Sodium citrate	2.00 g	1.40 g	-	-
Glucose	3.00 g	3.00 g	-	-
Citric acid	-	-	-	1.64 g
Fructose	-	-	2.50 g	2.00 g
Tris	-	-	-	3.07 g
Skimmed milk powder	-	-	10.00 g	-
Soy Lecithin	-	-	0.375 g	-
Egg yolk	10%	-	-	10%
Penicillin sodium	100,000 IU	100,000 IU	100,000 IU	100,000 IU
Streptomycin sulfate	100,000 IU	100,000 IU	100,000 IU	100,000 IU
Total volume	100 mL	100 mL	100 mL	100 mL
Osmotic pressure	377 m Osm/L	316 m Osm/L	410 m Osm/L	375 m Osm/L

**Table 2:** Effects of different diluents on the motility parameters of the *Hu* ram sperm.

Motility Parameters	Preserved Period (Days)	Different Diluents			
		A	B	C	D
TM (%)	0	94.40 ± 0.02 ^A^	94.95 ± 0.06 ^A^	94.85 ± 0.40 ^A^	94.11 ± 0.92 ^A^
	1	93.56 ± 0.14 ^Aa^	79.67 ± 0.24 ^Bc^	88.64 ± 0.43 ^Bb^	93.37 ± 0.41 ^ABa^
	2	91.74 ± 0.10 ^Ba^	74.28 ± 1.86 ^Cb^	37.84 ± 0.72 ^Cc^	91.31 ± 1.34 ^BCa^
	3	85.92 ± 0.73 ^Cb^	64.22 ± 1.31 ^Dc^	0 ^Dd^	90.11 ± 0.06 ^CDa^
	4	83.06 ± 0.10 ^Db^	49.90 ± 0.21 ^Ec^	0 ^Dd^	88.96 ± 0.38 ^Da^
	5	80.86 ± 1.11 ^Eb^	32.01 ± 0.30 ^Fc^	0 ^Dd^	86.44 ± 0.58 ^Ea^
	6	69.53 ± 0.36 ^Fb^	14.60 ± 0.31 ^Gc^	0 ^Dd^	80.53 ± 0.21 ^Fa^
	7	34.45 ± 0.47 ^Gb^	0 ^Hc^	0 ^Dc^	51.01 ± 0.69 ^Ga^
VSL (µm/s)	0	41.34 ± 0.57 ^AB^	41.36 ± 1.59 ^B^	42.05 ± 1.64 ^A^	41.15 ± 1.65 ^BC^
	1	42.98 ± 1.64 ^A^	38.66 ± 1.78 ^B^	39.65 ± 1.23 ^B^	43.02 ± 1.01 ^AB^
	2	45.31 ± 1.27 ^Aab^	47.77 ± 0.23 ^Aa^	26.98 ± 0.45 ^Cc^	44.70 ± 0.74 ^Ab^
	3	44.69 ± 2.02 ^Aa^	41.04 ± 2.61 ^Ba^	0 ^Db^	38.98 ± 0.66 ^CDa^
	4	41.17 ± 0.07 ^ABa^	43.23 ± 1.12 ^Ba^	0 ^Db^	43.00 ± 0.66 ^ABa^
	5	34.45 ± 0.20 ^Cb^	30.33 ± 0.35 ^Cc^	0 ^Dd^	37.26 ± 0.28 ^Da^
	6	36.33 ± 0.25 ^BCb^	19.70 ± 1.86 ^Dc^	0 ^Dd^	40.16 ± 1.03 ^BCa^
	7	35.33 ± 3.40 ^Ca^	0 ^Eb^	0 ^Db^	33.57 ± 0.13 ^Ea^
VCL (µm/s)	0	80.82 ± 0.98 ^A^	82.22 ± 0.08 ^A^	82.36 ± 2.62 ^A^	82.11 ± 0.86 ^A^
	1	75.71 ± 2.19 ^ABab^	69.84 ± 2.91 ^Bb^	72.23 ± 2.12 ^Bb^	81.09 ± 1.08 ^Aa^
	2	78.38 ± 1.71 ^Ab^	83.16 ± 1.23 ^Aa^	43.30 ± 1.13 ^Cc^	82.88 ± 1.22 ^Aa^
	3	74.32 ± 3.56 ^ABa^	69.48 ± 3.43 ^Ba^	0 ^Db^	75.85 ± 0.78 ^BCa^
	4	69.94 ± 0.45 ^BCb^	69.39 ± 1.15 ^Bb^	0 ^Dc^	78.62 ± 1.09 ^ABa^
	5	61.67 ± 0.85 ^Db^	52.06 ± 0.15 ^Cc^	0 ^Dd^	67.26 ± 0.47 ^Da^
	6	64.39 ± 0.26 ^CDb^	41.92 ± 2.68 ^Dc^	0 ^Dd^	73.76 ± 2.86 ^Ca^
	7	65.86 ± 5.14 ^CDa^	0 ^Eb^	0 ^Db^	62.93 ± 0.74 ^Ea^
VAP (µm/s)	0	57.15 ± 0.69 ^A^	58.14 ± 0.05 ^A^	58.23 ± 1.85 ^A^	57.97 ± 0.61 ^A^
	1	53.54 ± 1.54 ^ABab^	49.38 ± 2.06 ^Bc^	51.08 ± 1.50 ^Bb^	57.34 ± 0.77 ^Aa^
	2	55.42 ± 1.21 ^Ab^	58.80 ± 0.87 ^Aa^	30.62 ± 0.80 ^Cc^	58.61 ± 0.86 ^Aa^
	3	52.56 ± 2.51 ^ABa^	49.13 ± 2.43 ^Ba^	0 ^Db^	53.64 ± 0.55 ^BCa^
	4	49.46 ± 0.31 ^BCb^	49.07 ± 0.81 ^Bb^	0 ^Dc^	55.60 ± 0.77 ^ABa^
	5	43.61 ± 0.60 ^Db^	36.81 ± 0.10 ^Cc^	0 ^Dd^	47.56 ± 0.33 ^Da^
	6	45.53 ± 0.18 ^CDb^	29.64 ± 1.90 ^Dc^	0 ^Dd^	52.16 ± 2.02 ^Ca^
	7	46.57 ± 3.63 ^CDa^	0 ^Eb^	0 ^Db^	44.50 ± 0.53 ^Ea^
MAD (°/s)	0	133.19 ± 7.61 ^A^	133.19 ± 6.76 ^A^	132.37 ± 8.70 ^A^	132.50 ± 7.07 ^A^
	1	101.36 ± 5.65 ^Ba^	77.65 ± 2.43 ^Bb^	106.97 ± 11.17 ^Ba^	118.47 ± 5.20 ^Ba^
	2	98.55 ± 2.06 ^BCa^	74.15 ± 4.01 ^Bb^	28.66 ± 0.75 ^Cc^	107.33 ± 3.91 ^BCa^
	3	88.46 ± 1.52 ^BCDb^	55.34 ± 1.60 ^Cc^	0 ^Dd^	101.15 ± 3.07 ^CDa^
	4	88.00 ± 1.68 ^CDb^	40.07 ± 0.94 ^Dc^	0 ^Dd^	95.65 ± 0.87 ^CDa^
	5	81.92 ± 4.64 ^Db^	23.84 ± 0.58 ^Ec^	0 ^Dd^	93.60 ± 2.20 ^Da^
	6	59.36 ± 3.56 ^Eb^	11.65 ± 0.91 ^Fc^	0 ^Dd^	71.99 ± 0.50 ^Ea^
	7	28.59 ± 1.52 ^Fb^	0 ^Gc^	0 ^Dc^	34.18 ± 3.00 ^Fa^

Note: Data are expressed as mean ± SD of *Hu* ram sperm in different diluents. Different superscripts (lowercase) in the same row show significant differences (*p* < 0.05). Different superscripts (uppercase) in the same column show significant differences (*p* < 0.05).

**Table 3:** Effects of different diluents on the functional integrity of the *Hu* ram sperm.

Parameter	Preserved Period (Days)	Different Diluents			
		A	B	C	D
Sperm functional membrane integrity (%)	0	84.54 ± 0.59 ^A^	84.51 ± 0.64 ^A^	84.83 ± 0.63 ^A^	85.10 ± 0.21 ^A^
	1	73.65 ± 0.32 ^Bb^	64.30 ± 1.29 ^Bc^	65.77 ± 1.63 ^Bc^	77.44 ± 0.33 ^Ba^
	2	69.44 ± 0.76 ^Cb^	60.79 ± 2.10 ^Bc^	40.58 ± 1.66 ^Cd^	75.00 ± 0.73 ^Ca^
	3	63.47 ± 0.67 ^Db^	54.85 ± 0.43 ^Cc^	-	71.22 ± 0.35 ^Da^
	4	57.67 ± 0.60 ^Eb^	50.78 ± 0.29 ^Dc^	-	67.62 ± 0.87 ^Ea^
	5	52.32 ± 0.54 ^Fb^	44.92 ± 0.79 ^Ec^	-	61.26 ± 0.45 ^Fa^
	6	38.22 ± 0.36 ^Gb^	35.09 ± 0.38 ^Fc^	-	54.12 ± 1.46 ^Ga^
	7	34.23 ± 1.75 ^Hb^	-	-	45.38 ± 0.75 ^Ha^
Sperm acrosome integrity (%)	0	91.13 ± 0.23 ^A^	90.84 ± 0.33 ^A^	91.01 ± 0.56 ^A^	90.87 ± 0.43 ^A^
	1	89.83 ± 0.47 ^Ba^	82.70 ± 0.31 ^Bc^	84.53 ± 1.38 ^Bbc^	85.92 ± 0.60 ^Bb^
	2	80.75 ± 0.04 ^Cb^	75.19 ± 0.17 ^Cc^	83.08 ± 0.46 ^Ba^	84.41 ± 0.67 ^Ba^
	3	80.94 ± 0.64 ^Ca^	74.17 ± 1.02 ^Cb^	-	82.95 ± 0.74 ^Ca^
	4	79.11 ± 0.92 ^Ca^	73.61 ± 0.63 ^Cb^	-	79.48 ± 1.37 ^Da^
	5	70.26 ± 0.10 ^Db^	58.60 ± 1.43 ^Dc^	-	77.02 ± 0.47 ^Ea^
	6	57.65 ± 3.06 ^Ea^	42.72 ± 0.91 ^Eb^	-	64.13 ± 0.27 ^Fa^
	7	40.46 ± 0.88 ^Fb^	-	-	45.95 ± 0.63 ^Ga^

Note: Data are expressed as mean ± SD of *Hu* ram sperm in different diluents. Different superscripts (lowercase) in the same row show significant differences. (*p* < 0.05). Different superscripts (uppercase) in the same column show significant differences. (*p* < 0.05). “-” means that all the sperm had died, meaning loss of detection.

## Data Availability

All data sets collected and analyzed during the current study are available from the corresponding author on reasonable request.
